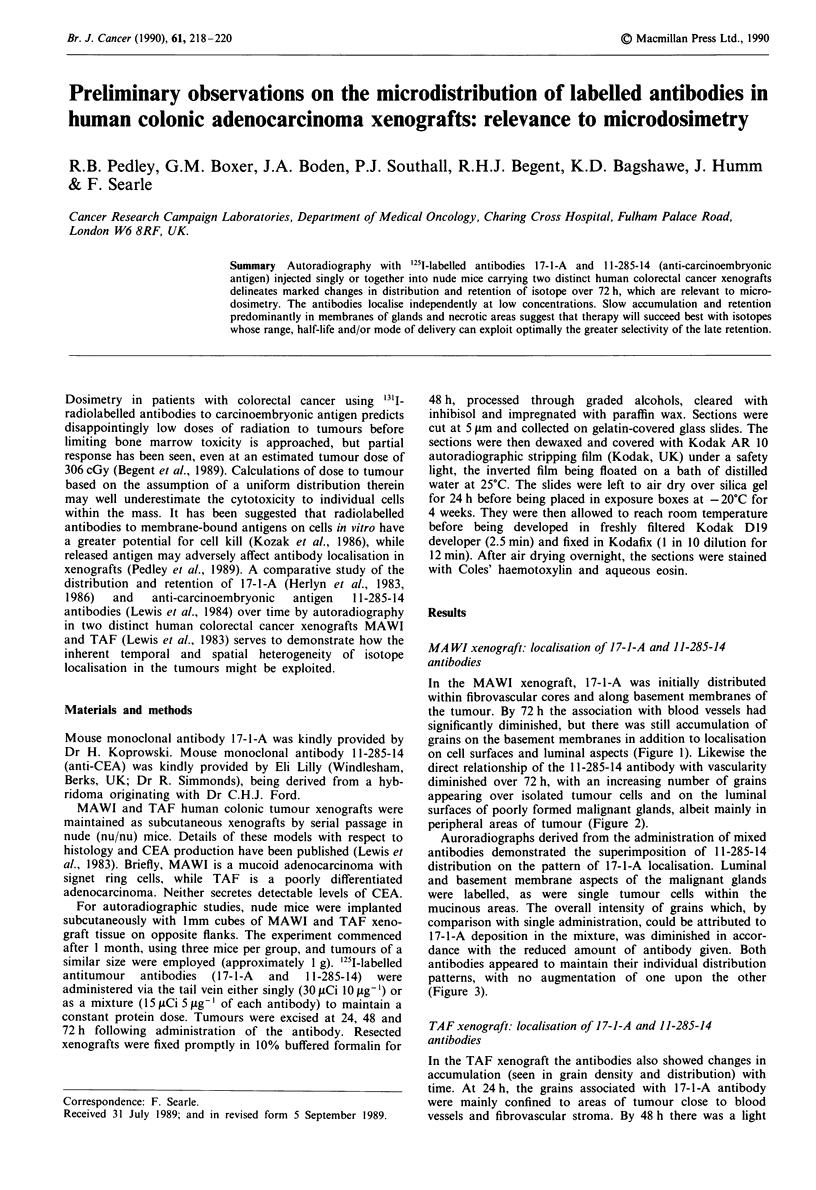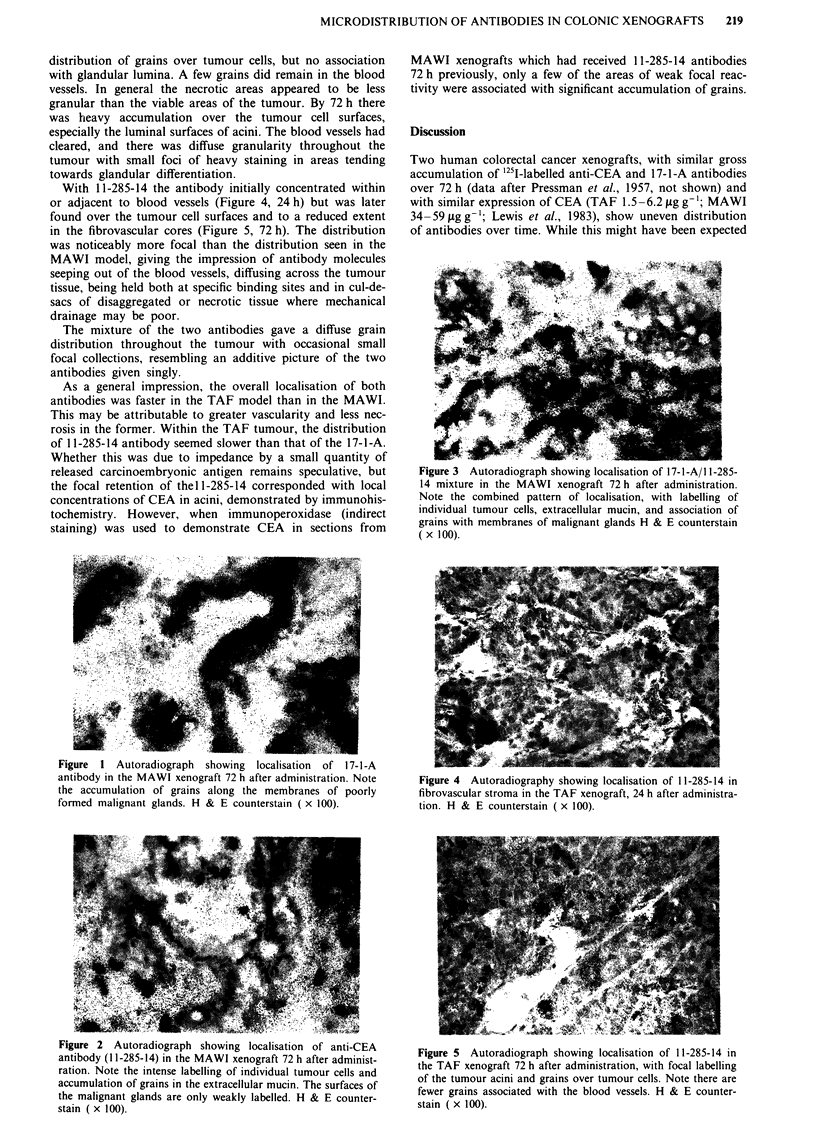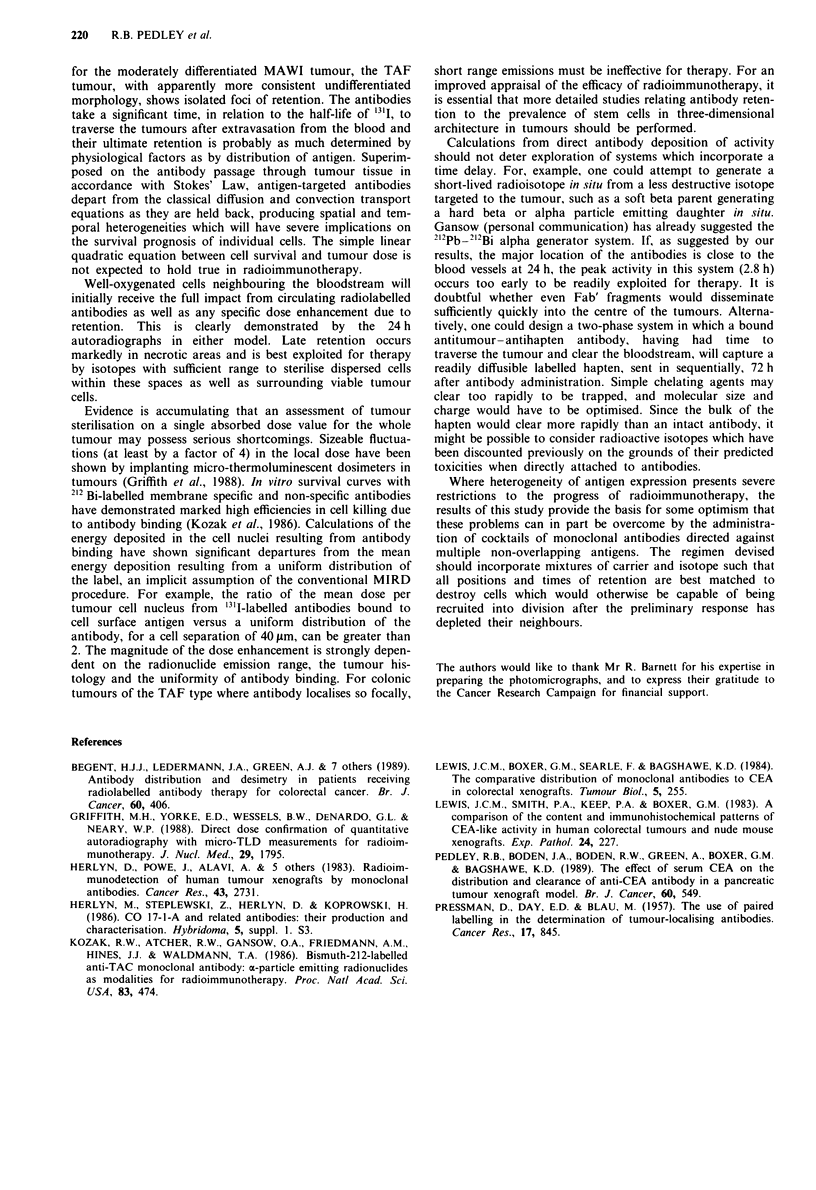# Preliminary observations on the microdistribution of labelled antibodies in human colonic adenocarcinoma xenografts: relevance to microdosimetry.

**DOI:** 10.1038/bjc.1990.40

**Published:** 1990-02

**Authors:** R. B. Pedley, G. M. Boxer, J. A. Boden, P. J. Southall, R. H. Begent, K. D. Bagshawe, J. Humm, F. Searle

**Affiliations:** Department of Medical Oncology, Charing Cross Hospital, London, UK.

## Abstract

**Images:**


*Br. J. Cancer* (1990), **61**, 218–220 © Macmillan Press Ltd., 1990

### Preliminary observations on the microdistribution of labelled antibodies in human colonic adenocarcinoma xenografts: relevance to microdosimetry

R.B. Pedley, G.M. Boxer, J.A. Boden, P.J. Southall, R.H.J. Begent, K.D. Bagshawe, J. Humm & F. Searle

*Cancer Research Campaign Laboratories, Department of Medical Oncology, Charing Cross Hospital, Fulham Palace Road, London W6 8RF, UK.*

**Summary** Autoradiography with $^{125}$I-labelled antibodies 17-1-A and 11-285-14 (anti-carcinoembryonic antigen) injected singly or together into nude mice carrying two distinct human colorectal cancer xenografts delineates marked changes in distribution and retention of isotope over 72 h, which are relevant to microdosimetry. The antibodies localise independently at low concentrations. Slow accumulation and retention predominantly in membranes of glands and necrotic areas suggest that therapy will succeed best with isotopes whose range, half-life and/or mode of delivery can exploit optimally the greater selectivity of the late retention.

Dosimetry in patients with colorectal cancer using $^{131}$I-radiolabelled antibodies to carcinoembryonic antigen predicts disappointingly low doses of radiation to tumours before limiting bone marrow toxicity is approached, but partial response has been seen, even at an estimated tumour dose of 306 cGy (Begent *et al.*, 1989). Calculations of dose to tumour based on the assumption of a uniform distribution therein may well underestimate the cytotoxicity to individual cells within the mass. It has been suggested that radiolabelled antibodies to membrane-bound antigens on cells *in vitro* have a greater potential for cell kill (Kozak *et al.*, 1986), while released antigen may adversely affect antibody localisation in xenografts (Pedley *et al.*, 1989). A comparative study of the distribution and retention of 17-1-A (Herlyn *et al.*, 1983, 1986) and anti-carcinoembryonic antigen 11-285-14 antibodies (Lewis *et al.*, 1984) over time by autoradiography in two distinct human colorectal cancer xenografts MAWI and TAF (Lewis *et al.*, 1983) serves to demonstrate how the inherent temporal and spatial heterogeneity of isotope localisation in the tumours might be exploited.

### Materials and methods

Mouse monoclonal antibody 17-1-A was kindly provided by Dr H. Koprowski. Mouse monoclonal antibody 11-285-14 (anti-CEA) was kindly provided by Eli Lilly (Windlesham, Berks, UK; Dr R. Simmonds), being derived from a hybridoma originating with Dr C.H.J. Ford.

MAWI and TAF human colonic tumour xenografts were maintained as subcutaneous xenografts by serial passage in nude (nu/nu) mice. Details of these models with respect to histology and CEA production have been published (Lewis *et al.*, 1983). Briefly, MAWI is a mucoid adenocarcinoma with signet ring cells, while TAF is a poorly differentiated adenocarcinoma. Neither secretes detectable levels of CEA.

For autoradiographic studies, nude mice were implanted subcutaneously with lmm cubes of MAWI and TAF xenograft tissue on opposite flanks. The experiment commenced after 1 month, using three mice per group, and tumours of a similar size were employed (approximately 1 g). $^{125}$I-labelled antitumour antibodies (17-1-A and 11-285-14) were administered via the tail vein either singly (30 $\mu$Ci 10 $\mu g^{-1}$) or as a mixture (15 $\mu$Ci 5 $\mu g^{-1}$ of each antibody) to maintain a constant protein dose. Tumours were excised at 24, 48 and 72 h following administration of the antibody. Resected xenografts were fixed promptly in 10% buffered formalin for 48 h, processed through graded alcohols, cleared with inhibisol and impregnated with paraffin wax. Sections were cut at 5 $\mu$m and collected on gelatin-covered glass slides. The sections were then dewaxed and covered with Kodak AR 10 autoradiographic stripping film (Kodak, UK) under a safety light, the inverted film being floated on a bath of distilled water at 25°C. The slides were left to air dry over silica gel for 24 h before being placed in exposure boxes at −20°C for 4 weeks. They were then allowed to reach room temperature before being developed in freshly filtered Kodak D19 developer (2.5 min) and fixed in Kodafix (1 in 10 dilution for 12 min). After air drying overnight, the sections were stained with Coles' haemotoxylin and aqueous eosin.

### Results

### *MAWI xenograft: localisation of 17-1-A and 11-285-14 antibodies*

In the MAWI xenograft, 17-1-A was initially distributed within fibrovascular cores and along basement membranes of the tumour. By 72 h the association with blood vessels had significantly diminished, but there was still accumulation of grains on the basement membranes in addition to localisation on cell surfaces and luminal aspects (Figure 1). Likewise the direct relationship of the 11-285-14 antibody with vascularity diminished over 72 h, with an increasing number of grains appearing over isolated tumour cells and on the luminal surfaces of poorly formed malignant glands, albeit mainly in peripheral areas of tumour (Figure 2).

Auroradiographs derived from the administration of mixed antibodies demonstrated the superimposition of 11-285-14 distribution on the pattern of 17-1-A localisation. Luminal and basement membrane aspects of the malignant glands were labelled, as were single tumour cells within the mucinous areas. The overall intensity of grains which, by comparison with single administration, could be attributed to 17-1-A deposition in the mixture, was diminished in accordance with the reduced amount of antibody given. Both antibodies appeared to maintain their individual distribution patterns, with no augmentation of one upon the other (Figure 3).

### *TAF xenograft: localisation of 17-1-A and 11-285-14 antibodies*

In the TAF xenograft the antibodies also showed changes in accumulation (seen in grain density and distribution) with time. At 24 h, the grains associated with 17-1-A antibody were mainly confined to areas of tumour close to blood vessels and fibrovascular stroma. By 48 h there was a light

Correspondence: F. Searle.
Received 31 July 1989; and in revised form 5 September 1989.

distribution of grains over tumour cells, but no association with glandular lumina. A few grains did remain in the blood vessels. In general the necrotic areas appeared to be less granular than the viable areas of the tumour. By 72 h there was heavy accumulation over the tumour cell surfaces, especially the luminal surfaces of acini. The blood vessels had cleared, and there was diffuse granularity throughout the tumour with small foci of heavy staining in areas tending towards glandular differentiation.

With 11-285-14 the antibody initially concentrated within or adjacent to blood vessels (Figure 4, 24 h) but was later found over the tumour cell surfaces and to a reduced extent in the fibrovascular cores (Figure 5, 72 h). The distribution was noticeably more focal than the distribution seen in the MAWI model, giving the impression of antibody molecules seeping out of the blood vessels, diffusing across the tumour tissue, being held both at specific binding sites and in cul-de-sacs of disaggregated or necrotic tissue where mechanical drainage may be poor.

The mixture of the two antibodies gave a diffuse grain distribution throughout the tumour with occasional small focal collections, resembling an additive picture of the two antibodies given singly.

As a general impression, the overall localisation of both antibodies was faster in the TAF model than in the MAWI. This may be attributable to greater vascularity and less necrosis in the former. Within the TAF tumour, the distribution of 11-285-14 antibody seemed slower than that of the 17-1-A. Whether this was due to impedance by a small quantity of released carcinoembryonic antigen remains speculative, but the focal retention of the11-285-14 corresponded with local concentrations of CEA in acini, demonstrated by immunohistochemistry. However, when immunoperoxidase (indirect staining) was used to demonstrate CEA in sections from MAWI xenografts which had received 11-285-14 antibodies 72 h previously, only a few of the areas of weak focal reactivity were associated with significant accumulation of grains.

### Discussion

Two human colorectal cancer xenografts, with similar gross accumulation of $^{125}$I-labelled anti-CEA and 17-1-A antibodies over 72 h (data after Pressman *et al.*, 1957, not shown) and with similar expression of CEA (TAF 1.5–6.2 $\mu g\ g^{-1}$; MAWI 34–59 $\mu g\ g^{-1}$; Lewis *et al.*, 1983), show uneven distribution of antibodies over time. While this might have been expected

**Figure 1** Autoradiograph showing localisation of 17-1-A antibody in the MAWI xenograft 72 h after administration. Note the accumulation of grains along the membranes of poorly formed malignant glands. H & E counterstain ( × 100).

**Figure 2** Autoradiograph showing localisation of anti-CEA antibody (11-285-14) in the MAWI xenograft 72 h after administration. Note the intense labelling of individual tumour cells and accumulation of grains in the extracellular mucin. The surfaces of the malignant glands are only weakly labelled. H & E counterstain ( × 100).

**Figure 3** Autoradiograph showing localisation of 17-1-A/11-285-14 mixture in the MAWI xenograft 72 h after administration. Note the combined pattern of localisation, with labelling of individual tumour cells, extracellular mucin, and association of grains with membranes of malignant glands H & E counterstain ( × 100).

**Figure 4** Autoradiography showing localisation of 11-285-14 in fibrovascular stroma in the TAF xenograft, 24 h after administration. H & E counterstain ( × 100).

**Figure 5** Autoradiograph showing localisation of 11-285-14 in the TAF xenograft 72 h after administration, with focal labelling of the tumour acini and grains over tumour cells. Note there are fewer grains associated with the blood vessels. H & E counterstain ( × 100).

for the moderately differentiated MAWI tumour, the TAF tumour, with apparently more consistent undifferentiated morphology, shows isolated foci of retention. The antibodies take a significant time, in relation to the half-life of $^{131}I$, to traverse the tumours after extravasation from the blood and their ultimate retention is probably as much determined by physiological factors as by distribution of antigen. Superimposed on the antibody passage through tumour tissue in accordance with Stokes' Law, antigen-targeted antibodies depart from the classical diffusion and convection transport equations as they are held back, producing spatial and temporal heterogeneities which will have severe implications on the survival prognosis of individual cells. The simple linear quadratic equation between cell survival and tumour dose is not expected to hold true in radioimmunotherapy.

Well-oxygenated cells neighbouring the bloodstream will initially receive the full impact from circulating radiolabelled antibodies as well as any specific dose enhancement due to retention. This is clearly demonstrated by the 24 h autoradiographs in either model. Late retention occurs markedly in necrotic areas and is best exploited for therapy by isotopes with sufficient range to sterilise dispersed cells within these spaces as well as surrounding viable tumour cells.

Evidence is accumulating that an assessment of tumour sterilisation on a single absorbed dose value for the whole tumour may possess serious shortcomings. Sizeable fluctuations (at least by a factor of 4) in the local dose have been shown by implanting micro-thermoluminescent dosimeters in tumours (Griffith *et al.*, 1988). *In vitro* survival curves with $^{212}$Bi-labelled membrane specific and non-specific antibodies have demonstrated marked high efficiencies in cell killing due to antibody binding (Kozak *et al.*, 1986). Calculations of the energy deposited in the cell nuclei resulting from antibody binding have shown significant departures from the mean energy deposition resulting from a uniform distribution of the label, an implicit assumption of the conventional MIRD procedure. For example, the ratio of the mean dose per tumour cell nucleus from $^{131}$I-labelled antibodies bound to cell surface antigen versus a uniform distribution of the antibody, for a cell separation of 40 $\mu$m, can be greater than 2. The magnitude of the dose enhancement is strongly dependent on the radionuclide emission range, the tumour histology and the uniformity of antibody binding. For colonic tumours of the TAF type where antibody localises so focally, short range emissions must be ineffective for therapy. For an improved appraisal of the efficacy of radioimmunotherapy, it is essential that more detailed studies relating antibody retention to the prevalence of stem cells in three-dimensional architecture in tumours should be performed.

Calculations from direct antibody deposition of activity should not deter exploration of systems which incorporate a time delay. For, example, one could attempt to generate a short-lived radioisotope *in situ* from a less destructive isotope targeted to the tumour, such as a soft beta parent generating a hard beta or alpha particle emitting daughter *in situ*. Gansow (personal communication) has already suggested the $^{212}$Pb–$^{212}$Bi alpha generator system. If, as suggested by our results, the major location of the antibodies is close to the blood vessels at 24 h, the peak activity in this system (2.8 h) occurs too early to be readily exploited for therapy. It is doubtful whether even Fab′ fragments would disseminate sufficiently quickly into the centre of the tumours. Alternatively, one could design a two-phase system in which a bound antitumour–antihapten antibody, having had time to traverse the tumour and clear the bloodstream, will capture a readily diffusible labelled hapten, sent in sequentially, 72 h after antibody administration. Simple chelating agents may clear too rapidly to be trapped, and molecular size and charge would have to be optimised. Since the bulk of the hapten would clear more rapidly than an intact antibody, it might be possible to consider radioactive isotopes which have been discounted previously on the grounds of their predicted toxicities when directly attached to antibodies.

Where heterogeneity of antigen expression presents severe restrictions to the progress of radioimmunotherapy, the results of this study provide the basis for some optimism that these problems can in part be overcome by the administration of cocktails of monoclonal antibodies directed against multiple non-overlapping antigens. The regimen devised should incorporate mixtures of carrier and isotope such that all positions and times of retention are best matched to destroy cells which would otherwise be capable of being recruited into division after the preliminary response has depleted their neighbours.

The authors would like to thank Mr R. Barnett for his expertise in preparing the photomicrographs, and to express their gratitude to the Cancer Research Campaign for financial support.

### References

BEGENT, H.J.J., LEDERMANN, J.A., GREEN, A.J. & 7 others (1989). Antibody distribution and desimetry in patients receiving radiolabelled antibody therapy for colorectal cancer. *Br. J. Cancer*, **60**, 406.

GRIFFITH, M.H., YORKE, E.D., WESSELS, B.W., DENARDO, G.L. & NEARY, W.P. (1988). Direct dose confirmation of quantitative autoradiography with micro-TLD measurements for radioimmunotherapy. *J. Nucl. Med.*, **29**, 1795.

HERLYN, D., POWE, J., ALAVI, A. & 5 others (1983). Radioimmunodetection of human tumour xenografts by monoclonal antibodies. *Cancer Res.*, **43**, 2731.

HERLYN, M., STEPLEWSKI, Z., HERLYN, D. & KOPROWSKI, H. (1986). CO 17-1-A and related antibodies: their production and characterisation. *Hybridoma*, **5**, suppl. 1. S3.

KOZAK, R.W., ATCHER, R.W., GANSOW, O.A., FRIEDMANN, A.M., HINES, J.J. & WALDMANN, T.A. (1986). Bismuth-212-labelled anti-TAC monoclonal antibody: α-particle emitting radionuclides as modalities for radioimmunotherapy. *Proc. Natl Acad. Sci. USA*, **83**, 474.

LEWIS, J.C.M., BOXER, G.M., SEARLE, F. & BAGSHAWE, K.D. (1984). The comparative distribution of monoclonal antibodies to CEA in colorectal xenografts. *Tumour Biol.*, **5**, 255.

LEWIS, J.C.M., SMITH, P.A., KEEP, P.A. & BOXER, G.M. (1983). A comparison of the content and immunohistochemical patterns of CEA-like activity in human colorectal tumours and nude mouse xenografts. *Exp. Pathol.* **24**, 227.

PEDLEY, R.B., BODEN, J.A., BODEN, R.W., GREEN, A., BOXER, G.M. & BAGSHAWE, K.D. (1989). The effect of serum CEA on the distribution and clearance of anti-CEA antibody in a pancreatic tumour xenograft model. *Br. J. Cancer*, **60**, 549.

PRESSMAN, D., DAY, E.D. & BLAU, M. (1957). The use of paired labelling in the determination of tumour-localising antibodies. *Cancer Res.*, **17**, 845.